# Modulation of serotonin signaling/metabolism by *Akkermansia muciniphila* and its extracellular vesicles through the gut-brain axis in mice

**DOI:** 10.1038/s41598-020-79171-8

**Published:** 2020-12-17

**Authors:** Rezvan Yaghoubfar, Ava Behrouzi, Fatemeh Ashrafian, Arefeh Shahryari, Hamid Reza Moradi, Samira Choopani, Shima Hadifar, Farzam Vaziri, Seyed Ali Nojoumi, Abolfazl Fateh, Shohreh Khatami, Seyed Davar Siadat

**Affiliations:** 1 Department of Mycobacteriology and Pulmonary Research, Pasteur Institute of Iran, Tehran, Iran; 2 Microbiology Research Center (MRC), Pasteur Institute of Iran, Tehran, Iran; 3 Department of Basic sciences, School of Veterinary Medicine, Shiraz University, Shiraz, Iran; 4 Department of Physiology and Pharmacology, Pasteur Institute of Iran, Tehran, Iran; 5 Department of Biochemistry, Pasteur Institute of Iran, Tehran, Iran

**Keywords:** Cytokines, DNA, Biochemistry, Immunology, Microbiology, Neurology

## Abstract

Several studies have reported that the host-microbe interactions in the gut modulate the host serotonin or 5-hydroxytryptamine (5-HT) system. Here, we evaluated the effects of *Akkermansia muciniphila* and its extracellular vesicles (EVs) on genes pertaining to the serotonergic system in the colon and hippocampus of mice. Male C57BL/6J mice were administered viable *A. muciniphila* and its EVs for 4 weeks. The serotonin levels in the colon, hippocampus, and serum of mice, as well as the human colon carcinoma cells (Caco-2), were measured by ELISA assays. Also, the effects of *A. muciniphila* and its EVs on the expression of serotonin system genes in the colon and hippocampus were examined. *A. muciniphila* and its EVs may have a biological effect on the induction of serotonin levels in the colon and hippocampus of mice. Also, EVs increased the serotonin level in the Caco-2 cell line. In contrast, both treatments decreased the serotonin level in the serum. Both the bacterium and its EVs had significant effects on the mRNA expression of genes, involved in serotonin signaling/metabolism in the colon and hippocampus of mice. Moreover, *A. muciniphila* and its EVs affected the mRNA expression of inflammatory cytokines (*Il-10* and *Tnf*-α) in the colon, however, there is no significant difference in inflammatory cell infiltrate in the histopathology of the colon. The presence of *A. muciniphila* and its EVs in the gut promotes serotonin concentration, they also affect serotonin signaling/metabolism through the gut-brain axis and may be considered in new therapeutic strategies to ameliorate serotonin-related disorders.

## Introduction

The human gastrointestinal (GI) tract has a complex bacterial community, which regulates the host production of several signaling molecules, including serotonin or 5-hydroxytryptamine (5-HT), hormones, and neurotransmitters^1–3^. Serotonin is a biogenic amine with a wide variety of functions in the human body^4^. Considering the significant role of this neurotransmitter in the pathogenesis of GI and neurological disorders, there has been a growing interest in finding strategies for the treatment or prevention of GI and neuropsychiatric disorders^4,5^.

Serotonin, produced by enterochromaffin cells (ECCs) in the GI tract, accounts for approximately 95% of total serotonin in the body, while about 5% of the remaining serotonin is found in the brain^6–8^. Since GI is an important reservoir of serotonin, interactions between the microbiota and the serotonergic system play a fundamental role in the pathogenesis of gut disorders through the serotonin-gut microbiota axis. In this regard, a published study showed that the gut microbes crucially affect the production of colonic serotonin, given the effect of short-chain fatty acids (SCFAs) on enterochromaffin cells; this finding indicates the importance of the gut microbiota in the regulation of serotonergic system in the host^7^.

The intestinal microbiota can affect the serotonin concentration in the GI tract. It can also influence the host serotonergic neurotransmission in the gut-brain axis through neural processes between the enteric nervous system (ENS) and the central nervous system (CNS)^9–12^. The gut microbiota dysbiosis and dysfunction of the serotonergic system contribute to inflammatory bowel disease (IBD) and psychiatric disorders, such as mood disorders^13^, anxiety disorders^13^, depression^13^, and other systemic disorders^14^. There is a correlation between the gut microbiota and mucosal, neuronal, and systemic homeostasis of serotonin. Therefore, manipulation of the gut microbiota with probiotics can improve the serotonin system homeostasis and prevent several pathophysiological conditions in the host^15^.

In recent decades, many studies have focused on new probiotics to reach a better understanding of the properties and mechanisms of the next generation of probiotics in the prevention and treatment of several diseases^15,16^. *Akkermansia muciniphila* is a Gram-negative anaerobic bacterium, which can be abundantly found in the colon of healthy individuals. It constitutes up to 5% of the total microbiota population^17^ and plays an important role in the maintenance of GI tract homeostasis and the gut barrier integrity. Additionally, its abundance is inversely correlated with several diseases, such as IBD, colitis, and Crohn’s disease^18–21^.

Alongside classic microbe-associated molecular pattern (MAMP)-pattern recognition receptor (PRR) interactions between microbes and the host cell, metabolites derived from the gut microbiota, such as extracellular vesicles (EVs), can also serve as ligands for host receptors; therefore, their activities can affect the physiology and systemic metabolism of host cells^22^. The gut microbiota, along with other bacteria, also produce EVs^23,24^. Our recent laboratory study showed that *A. muciniphila*-derived EVs regulated the intestinal immunity and maintenance of immune homeostasis in the gut better than the bacterium itself^25^. However, little is known about the potential of these EVs.

Several studies have recently reported that EVs released by bacteria can cross the inner mucus layer, enter blood circulation, and pass across the blood–brain barrier (BBB) to the brain; consequently, they can influence the regulation of various pathways^26–30^. Given the role of EVs in the signaling processes of the gut-brain axis, they can be used in the peripheral and central serotonin pathways and contribute to both GI and neurological disorders. Therefore, in this study, we hypothesized that *A. muciniphila*-derived EVs might influence the serotonergic system in both the gut and the brain.

Since recent studies have highlighted the relationship between the gut microbiota and the serotonergic system, in this study, for the first time, we aimed to evaluate the effects of *A. muciniphila* and its EVs on the serotonergic system (i.e., synthesis, release, and clearance) in different anatomical regions of mice (e.g., colon and hippocampus) and also in human colon carcinoma cells (Caco-2 cell line).

## Materials and methods

### *A. muciniphila* culture and EV preparation

The *A. muciniphila* strain ATCC BAA-835, provided by the DSMZ Institute (German Collection of Microorganisms and Cell Cultures), was used in this study. The bacterium was routinely grown in a basal mucin-based medium under anaerobic conditions (80% N_2_, 10% H_2_, and 10% CO_2_) at 37 °C for 3–7 days^17^. The bacterium was routinely inoculated in brain–heart infusion (BHI) broth (Quelab, Canada), supplemented with 0.5% mucin (Sigma-Aldrich) under anaerobic conditions (150 rpm shaking) overnight at 37 °C until an optical density (OD_600_) of one was reached.

The bacterial cells were pelleted by centrifugation at 11,000×*g* for 20 min at 4 °C and washed twice with phosphate-buffered saline (PBS). Next, the suspended pellet was immediately placed on ice and used for cell culture treatment and oral administration in mice. We used the remaining supernatant to extract EVs. For this purpose, the supernatant was filtered through a 0.22-mm filter (Millipore, USA). The extraction of EVs was performed via ultracentrifugation at 200,000×*g* for two hours at 4 °C, as previously described^30^. The vesicle pellet was resuspended in sterile PBS and stored at − 80 °C for further use. The morphology of the isolated EVs was examined, using transmission electron microscopy (TEM).

### Animal experiments

Animal research was reviewed and approved by the Animal Experiment Committee of the Pasteur Institute of Iran (IR.PII.REC.1399.004), and confirming that all experiments were performed in accordance with relevant guidelines and regulations.

Thirty adult C57BL/6J male mice (age: 8 weeks) were purchased from Pasteur Institute of Iran (Karaj, Iran) and maintained under similar conditions (12:12 h light/dark cycle, 22–23 °C, and 40% humidity), with ad libitum access to food and autoclaved water.

### Experimental design

In this study, following oral gavage with *A. muciniphila* and EVs, the effects of both bacterium and EVs were evaluated in 30 mice (10 mice per group). The mice were fed a standard normal diet (A03, Safe Diet, France) over 1 week for acclimatization. Next, they were randomly divided into three groups: (1) receiving 200 μL of PBS (PBS or control group); (2) receiving 10^9^ colony-forming units (CFU)/200 μL of viable *A. muciniphila,* suspended in PBS (*A. muciniphila* group); and (3) 10 μg of protein/200 μL of EVs (EV group), once daily for 4 weeks. The body weight of each group was measured once a week.

### Sample collection

After 4 weeks of treatmen, all mice were sacrificed via cervical dislocation, and their blood samples and biopsies (organ collection) were collected. After the colon content was emptied, the tissue segments were collected in RNase-free Eppendorf tubes, snap-frozen with liquid nitrogen, and stored at − 80 °C for subsequent analyses. Next, the brain was rapidly removed from the cranium and the hippocampus was dissected in an ice-cold plate. Afterward, the tissue was flash-frozen in liquid nitrogen and kept at − 80 °C. The collected blood sample from the tail vein was centrifuged at 2500×*g* for ten minutes at 4 °C, and the serum samples were separated and stored at − 80 °C.

### Serotonin measurement

The serotonin level was quantified in the sera and supernatants of homogenized tissues, such as the colon and hippocampus. The tissues were weighed and then homogenized via ultrasonication, using the MSE Soniprep 150 Plus disintegrator in appropriate volumes of phenylmethylsulfonyl fluoride (PMSF; Sigma-Aldrich, Catalog Number: P7626), as a general inhibitor of a serine protease. The serotonin concentration was determined, using an ELISA kit (KA2518, Abnova, Taipei, Taiwan), according to the manufacturer’s protocols.

### Histological evaluation

After the colon samples were fixed in 10% formalin and embedded in paraffin, they were sectioned (5 mm), stained with hematoxylin and eosin (H&E), and observed under a light microscope^31^. Besides the effects of *A. muciniphila* and its EVs on the pathology of colon tissues, we investigated the effects of *A. muciniphila* and its EVs on the expression of inflammatory genes (*Tnf*-α and *Il-10*).

### Analysis of tissue gene expression

The RNA isolation and purification from tissues were according to the protocol of Ashrafian et al.^25^, In brief, the tissues were homogenized in a Precellys 24 homogenizer, using TRIzol reagent (Category No.: BS410, Bio Basic, Canada), and subsequently used for the purification of RNA. Then, total RNA was extracted according to the manufacturer’s instructions. RNA was treated with DNase-I (Qiagen, Germany) to remove genomic DNA. Following that, total RNA (500 ng) was used to synthesize cDNA in a PrimeScript RT Reagent Kit (Takara, USA). Quantitative RT-PCR measurements were performed using SYBR Premix Ex Taq II (Takara, USA). The oligonucleotide primer sequences used in this study are shown in Supplementary table.

### Cell culture conditions

The Caco-2 cells (ATCC HTB37) were obtained from the National Cell Bank of Iran (Pasteur Institute of Iran, Tehran), cultured at 37 °C in an atmosphere of 5% CO_2_, and maintained in high-glucose Dulbecco’s Modified Eagle’s Medium (HDMEM; Gibco, UK), supplemented with 1% non-essential amino acids, 1% penicillin/streptomycin (Gibco, UK), and 20% heat-inactivated fetal bovine serum (FBS; Gibco, UK) in six-well plates (Sorfa, China), as previously described^25^. Mycoplasma contamination was also assessed using a PCR assay.

### Cell culture treatment

For this purpose, the medium was changed every 2–3 days, and the cells were passaged enzymatically with a trypsin–EDTA solution 0.25% (0.53 mM). Next, they were subcultured in 75 cm^2^ plastic culture dishes (SPL Life Sciences Co., Korea). The Caco-2 cells were seeded in six-well plates (Sorfa, China) at a seeding density of 250,000 cells. After 14 days (ten days after confluence), the Caco-2 monolayer was infected with *A. muciniphila* at a multiplicity of infection (MOI) ratio of 100 (100 bacteria per cell)*,* as well as its EVs at a dose of 10 μg. We examined three MOIs of *A. muciniphila* and three doses of EVs; the best results were obtained at MOI of 100 and EV dose of 10 μg (data not shown). The duration of treatment was 24 h, and all of the experiments were performed in triplicate. An equal volume of PBS was administered as the control.

### RNA extraction, cDNA synthesis, and quantitative RT-PCR assay

After 24 h, the total RNA of the treated cells was extracted, using the RNeasy Plus Mini Kit (Qiagen, USA, Cat. No./ID: 74134), as previously described^25^. Next, cDNA synthesis was performed, using the PrimeScript RT Reagent Kit (Takara, Japan, Cat. No.: RR037A), according to the manufacturer’s instructions. Quantitative RT-PCR assay was also performed, using SYBR Premix Ex Taq II (Takara, USA) in triplicate. All primer sequences used in this study are shown in Supplementary table.

### Statistical analysis

The ΔΔCT method was used for analyzing the relative expression of *Gapdh* gene in the cell line, as well as *Rpl-13A* and *β-actin* genes in the tissue of mice as the endogenous control. The statistical analysis for Elisa assays and cycle threshold (CT) values analysis to calculate changes in gene expression were performed by GraphPad Prism 8.0 (GraphPad Software Inc, CA, and USA). The statistical significance of differences in the expression of target genes and Elisa assays was analyzed using one-way ANOVA. P-value of less than 0.05 was considered statistically significant.

## Results

### Characterization and morphology of *A. muciniphil*a-derived EVs

**Figure 1:**
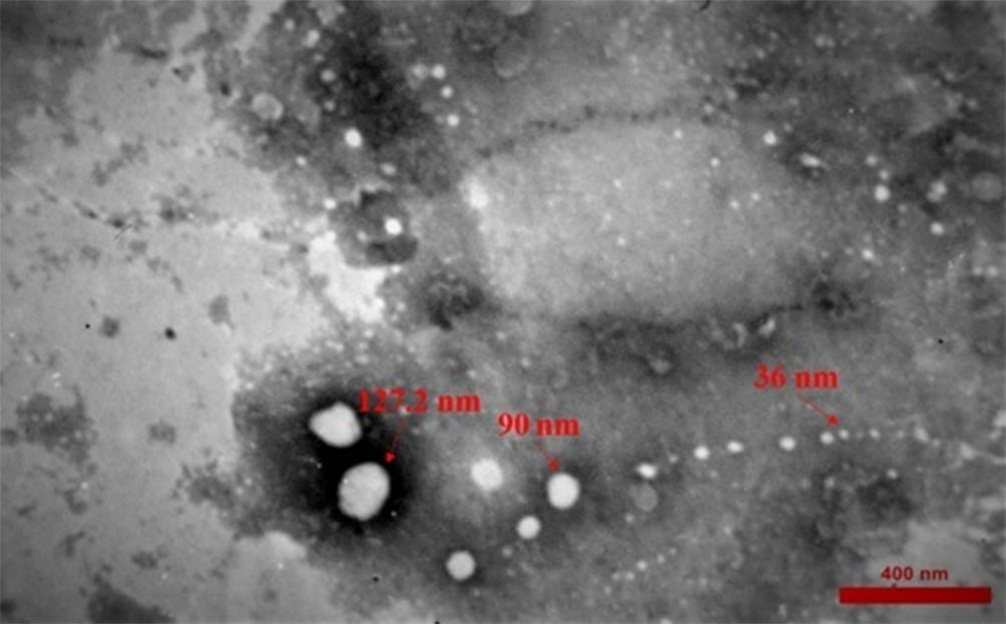
Morphological characterization of *A. muciniphila*-derived EVs. The TEM image of negatively stained EVs indicated different sizes (range: 30–150 nm) and vesicle-like structure of EVs (scale bar: 400 nm).

The extracted EVs from *A. muciniphila* were visualized by TEM. Multiple spherical vesicles were observed, the majority of which ranged from 40 to 150 nm in size (Fig. 1).

### Effects of *A. muciniphila* and its EVs on body weight, serotonin level in the colon, hippocampus, and serum of mice

**Figure 2:**
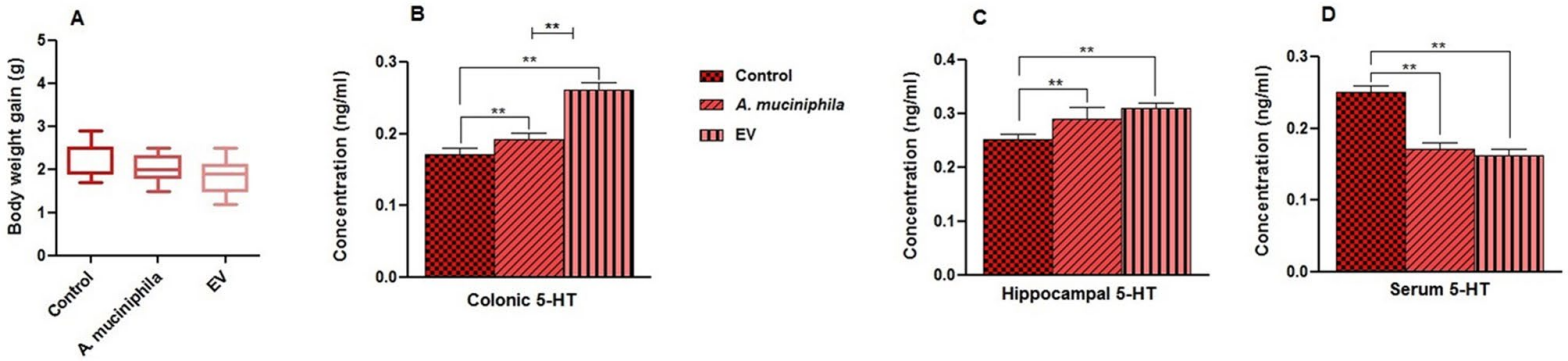
Effects of treatment with *A. muciniphila* and its EVs on the body weight and level of serotonin. This figure indicates the body weight (**A**) and level of serotonin released from (**B**) colon, (**C**) hippocampus, and (**D**) serum of mice. Data are standardized relative to the expression level of the control group (*P < 0.05 and **P < 0.01 are considered statistically significant).

In order to determine the effect of *A. muciniphila* and its EVs on serotonin system in mice, C57BL/6J ones were treated with *A. muciniphila* and its EVs every day for 4 weeks. Subsequently, the body weight of each group was measured once a week. The results showed that, there were no significant differences in the body weight between different treatment groups and the control group (Fig. 2A).

In the next stage, to assess the effect of *A. muciniphila* and its EVs on the serotonin overflow, we performed an ELISA assay to determine whether the oral gavage of *A. muciniphila* and its EVs altered the level of serotonin in the colon tissue, hippocampus, and serum of mice. There were differences in biological effects between the *A. muciniphila* and EV groups, compared to their control groups. The *A. muciniphila* (P = 0.003) and EV (P = 0.0001) groups showed a significant increase in the serotonin level of colon tissue, while EVs had more significant effects than the bacterium (Fig. 2B). Therefore, similar to the clone, both *A. muciniphila* (P = 0.0007) and EV (P = 0.0001) increased the serotonin level in the hippocampus (Fig. 2C). Also, the serum serotonin level was lower in the *A. muciniphila* (P = 0.0001) and EV (P = 0.0001) groups, compared to the control group (Fig. 2D). Overall, our results showed that EVs had more biological effects on increasing the serotonin level in various organs, compared to the bacterium itself. This probably suggests that EVs are more accessible to the cell than the bacterium itself and stimulate serotonin synthesis.

### *A. muciniphila* and its EVs induced changes in the expression of serotonergic genes in the colon of mice

**Figure 3:**
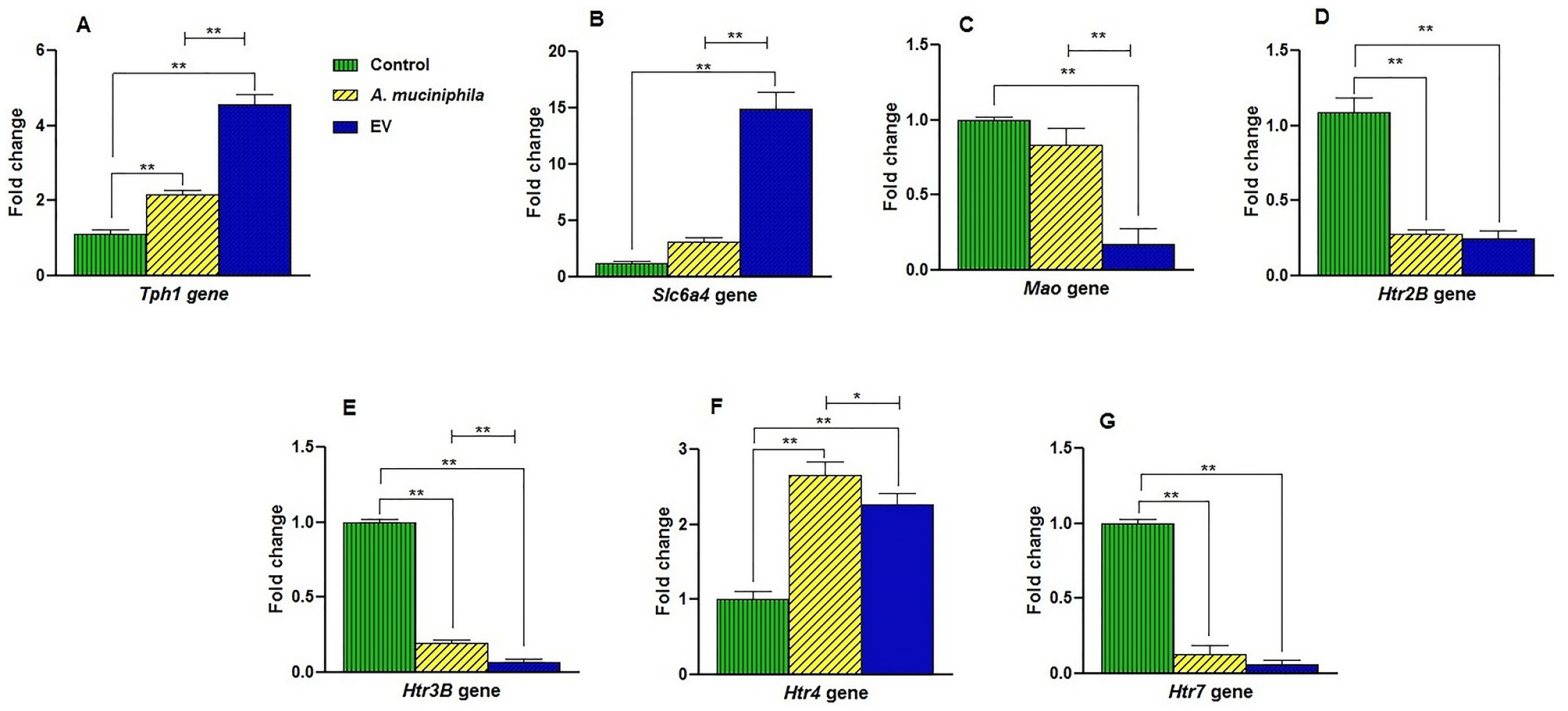
The effects of treatment with *A. muciniphila* and its EVs on the expression level of genes involved in the peripheral serotonergic system in the colon of mice. After 4 weeks of treatment, the relative mRNA expression in the colonic tissue of mice was affected by both the bacterium and its EVs. Expression of (**A**) *Tph1* (**B**) *Slc6a4* (**C**) *Mao* (**D**) *Htr2B* (**E**) *Htr*3B (**F**) *Htr*4, and (**G**) *Htr*7 genes. Data are normalized using *RPL13a* and *β-actin* as control genes. *, **, 'P < 0.05 and P < 0. 01 was considered statistically significant, respectively.

Given the effect of *A. muciniphila* and its EVs on the serotonin level, we investigated the expression of the following genes, involved in intestinal serotonin signaling: *Slc6a4* (involved in serotonin transport); *Tph1* (associated with the biosynthesis of serotonin); *Mao* (associated with serotonin metabolism); and *Htr2B*, *Htr3*, *Htr4,* and *Htr7* (receptors for serotonin). Both *A. muciniphila* and EV treatments significantly enhanced the gene expression of *Tph1* (P = 0.0014 and P = 0.0001, respectively) (Fig. 3A). Following treatment with the EV *Slc6a4* (P = 0.001) gene, it significantly increased when compared to the control group, whereas the *A. muciniphila* group did not show a significant impact on *Slc6a4* gene (Fig. 3B). Considering the effects of *A. muciniphila* and its EVs on two stages of the serotonergic system (i.e., synthesis and clearance), we appraised their effects on the metabolism of serotonin. These experiments showed that the expression of the *Mao* gene significantly reduced in the EV group (P = 0.0001) (Fig. 3C), compared to the control group, whereas the *A. muciniphila* group did not show a significant response to the treatment.

Following treatment with the bacterium, three genes showed a significant reduction in the expression of *Htr2B*, *Htr3,* and *Htr7* genes, compared to the control group (P-value *Htr*2B = 0.0001, *Htr3* = 0.0001, *Htr7* = 0.0001), respectively (Fig. 3D,E,G). In comparison with the control group, EV treatments significantly enhanced the expression of *Htr2B*, *Htr3,* and *Htr7* genes, (P-value *Htr*2B = 0.0001, *Htr3* = 0.0001, *Htr7* = 0.0001), respectively (Fig. 3D,E,G).

EVs induced a more significant decrease in *Htr3* expression, compared to the bacterium itself (Fig. 3E). On the contrary, the bacterium induced a more significant increase in the expression of *Htr4*, compared to EVs (P = 0.0001; Fig. 3F). These results indicate that *A. muciniphila* and its EVs affected the expression of genes involved in the colon serotonergic system of mice.

### Administration of *A. muciniphila* and its EVs affected the mRNA expression of genes involved in the serotonergic system in the hippocampus of mice

**Figure 4:**
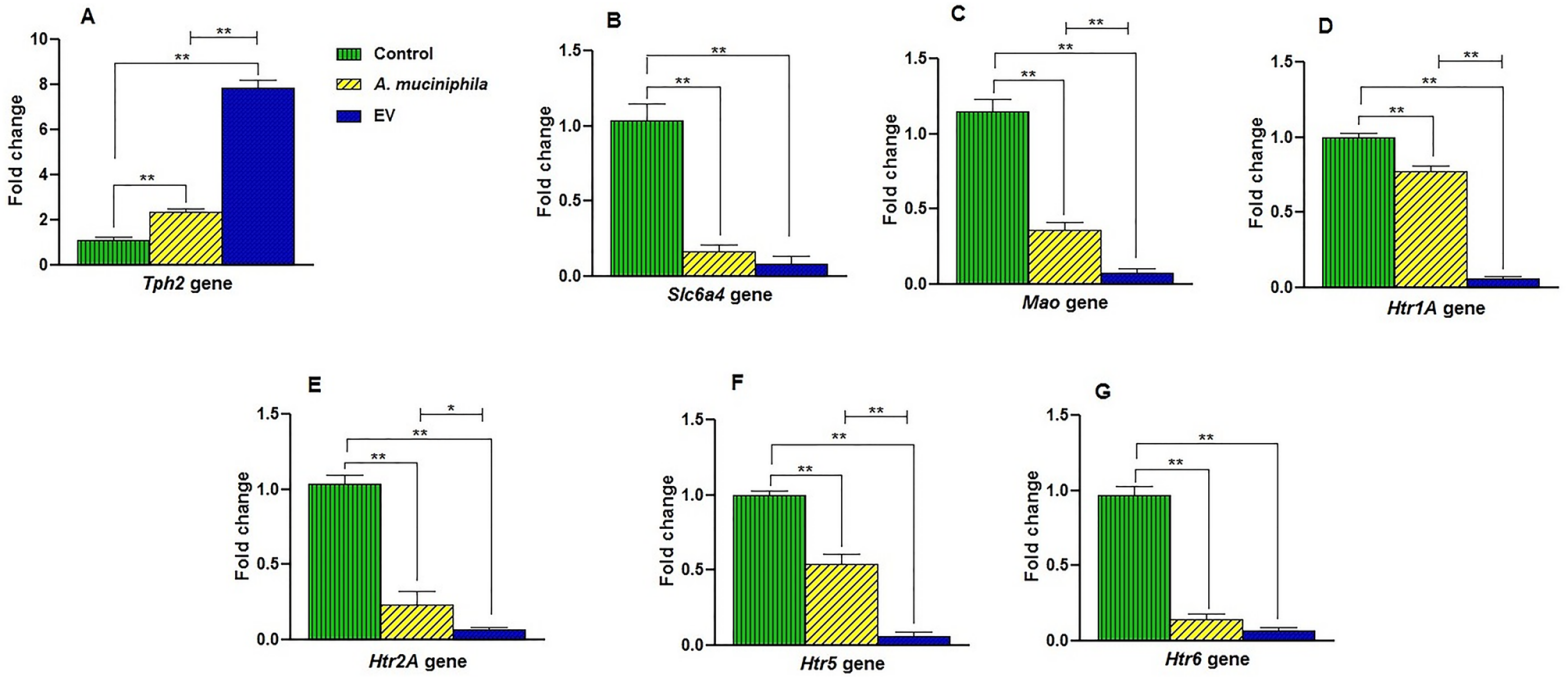
Administration of *A. muciniphila* and its EVs induced changes in the expression of hippocampal serotonergic system-related gene in mice. Expression of (**A**) *Tph2* (**B**) *Slc6a4* (**C**) *Mao* (**D**) *Htr1A* (**E**) *Htr2A* (**F**) *Htr5*, and (**G**) *Htr6* genes. Data are normalized using *RPL13a* and *β-actin* as control genes. *, **, 'P < 0.05 and P < 0. 01 were considered statistically significant, respectively.

To investigate the effects of *A. muciniphila* and its EVs on the hippocampal serotonergic system, the expression of *Tph2, Slc6a4, Mao, Htr1A, Htr2A, Htr5,* and *Htr6* genes was evaluated. After treatment, an increase in the expression level of the *Tph2* gene was observed in both *A. muciniphila* (P = 0.001) and EV (P = 0.0001) groups (Fig. 4A). In comparison with *A*. *muciniphila,* EVs induced a higher level of *Tph2* gene expression in the hippocampus of mice (Fig. 4A). The mRNA expression of *Slc6a4* (P = 0.0001 and P = 0.0001, respectively) (Fig. 4B) and *Mao* (P = 0.0001 and P = 0.0001, respectively) (Fig. 4C) genes significantly decreased in the bacterium and EV groups. Also, EV treatment induced lower levels of *Mao* expression*,* compared to the *A. muciniphila* group (Figs. 4D–F).

In response to treatment with *A. muciniphila* and its EVs, the mRNA expression of genes, including *Htr1A, Htr2A, Htr5,* and *Htr6,* significantly reduced in both *A. muciniphila* (P = 0.0002, P = 0.0001, P = 0.0001, P = 0.0001) (Fig. 4D–G) and EV groups (P = 0.0001, P = 0.0001, P = 0.0001, P = 0.0001) (Fig. 4D–G). The EVs induced lower levels of *Htr1A, Htr2A,* and *Htr5* expression*,* compared to the *A. muciniphila* group (Fig. 4D-G). These results showed that the administration of *A. muciniphila* and its EVs changed the expression of genes involved in the hippocampus serotonergic system of mice. Also, the gene expression analyses indicated that interactions of *A. muciniphila* and EVs with the host could affect the serotonergic system in the brain.

### *A. muciniphila* and its EVs modulated the serotonergic system in the colon of mice without causing adverse effect on inflammation

**Figure 5:**
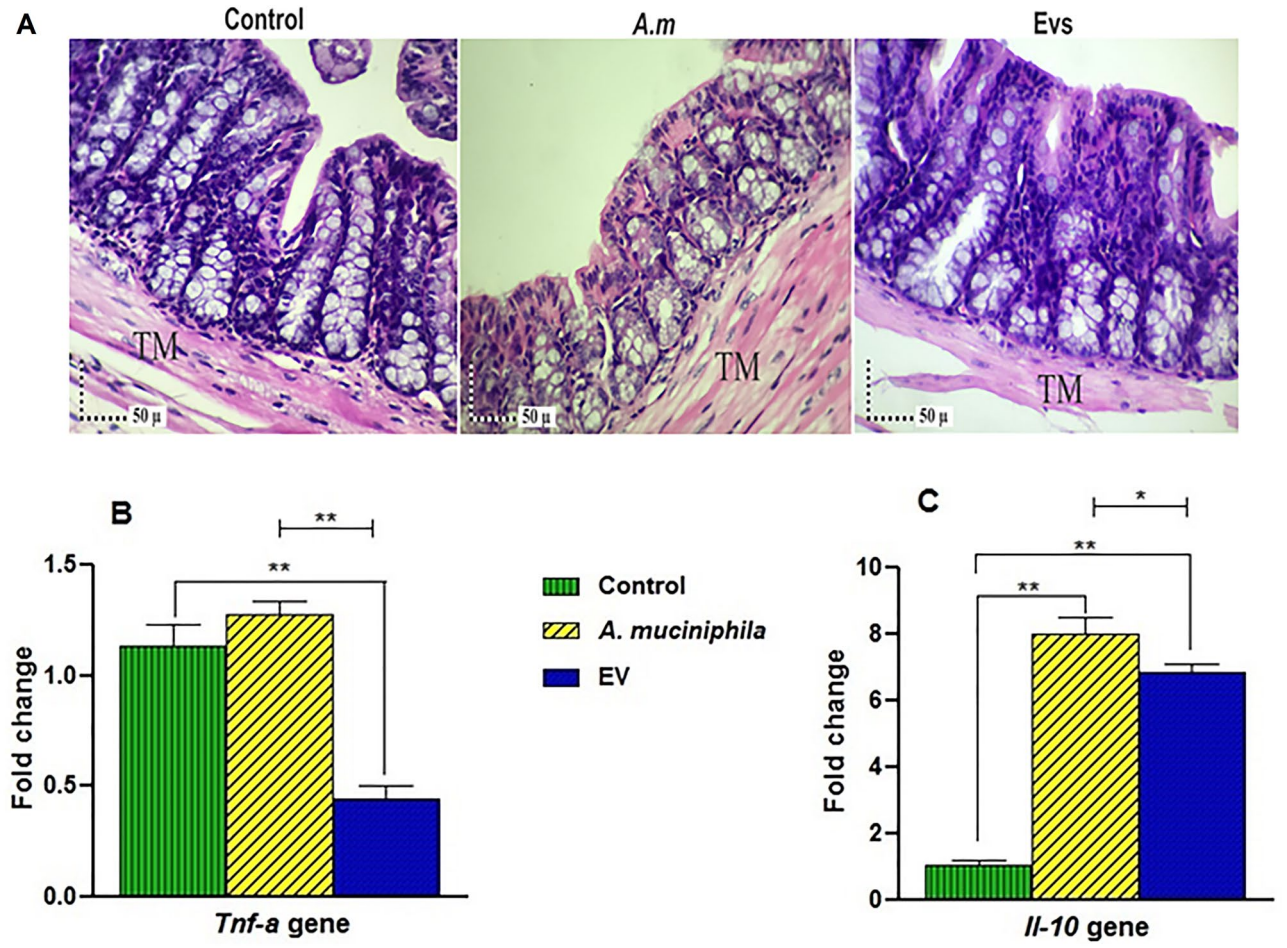
Administration of *A. muciniphila* and its EVs had an inverse correlation with colonic inflammation. (**A**) The difference in the histomorphology of the colon is evident in the H&E-stained sections of different groups. In groups receiving EVs and *A. muciniphila*, no significant difference in inflammatory cell infiltrate could be demonstrated in the mucosal (epithelium and lamina propria) and submucosal layers of the colon. The tunica muscularis of the colon was also observed; (**B**) expression of *Tnf-α* gene; and (**C**) expression of *Il-10* gene. Data are normalized using *RPL13a* and *β-actin* as control genes. *, **, 'P < 0.05 and P < 0.01 were considered statistically significant, respectively.

Since serotonin production can influence the process and severity of inflammation within the gut, we assessed the effects of *A. muciniphila* and its EVs on colonic inflammation in mice. In groups receiving the bacterium and its EV, no inflammatory cell infiltration was observed in the mucus, epithelium, lamina propria, and submucosal layers of the colon. These results indicated that the administration of *A. muciniphila* and its EVs increased serotonin levels; however it seems that they may not cause inflammation in the colon tissue (Fig. 5A).

Administration of *A. muciniphila* increased *Tnf-α* gene expression, although the difference was not statistically significant (Fig. 5B). In contrast, the EVs significantly reduced the expression of *nf-α* genes, compared to the control group (P = 0.0001; Fig. 5B). The mRNA expression of the *Il-10* gene was significantly higher in the treatment groups compared to the control group (P = 0.0001 and P = 0.0001, respectively) (Fig. 5C). Meanwhile, in comparison with EVs, *A. muciniphila* induced higher levels of *Il-10* gene expression in mice.

### Treatment with *A. muciniphila* and its EVs affected the in vitro serotonin release and serotonin-related genes in the Caco-2 cell line

**Figure 6:**
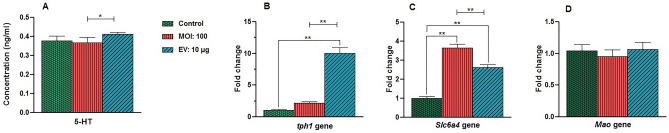
In vitro serotonin, availability was regulated by *A. muciniphila* and its EVs. The monolayer Caco-2 cell line was exposed to (**A**) *A. muciniphila* (MOI100) and EVs (10 μg) for 24 h. The figure represents the expression of (**B**) *Tph1*, (**C**) *Slc6a4*, and (**D**) *Mao* genes. The gene expression data are normalized relative to the GAPDH gene as the control (*P < 0.05 and **P < 0.01 indicated statistical significance).

To investigate the direct effects of *A. muciniphila* and its EVs on the serotonin level and serotonin-related genes in the serotonergic system, we used human epithelial colorectal adenocarcinoma cells (Caco-2 cell line) as a model to represent intestinal cells. After treatment with *A. muciniphila* (MOI_100_), the level of serotonin was not significantly different from the control group (Fig. 6A). In contrast, in the monolayer Caco-2 cell line, following exposure to EVs (10 μg), the level of serotonin increased while there was no significant difference in comparison with the controls (Fig. 6A).

**Figure 7:**
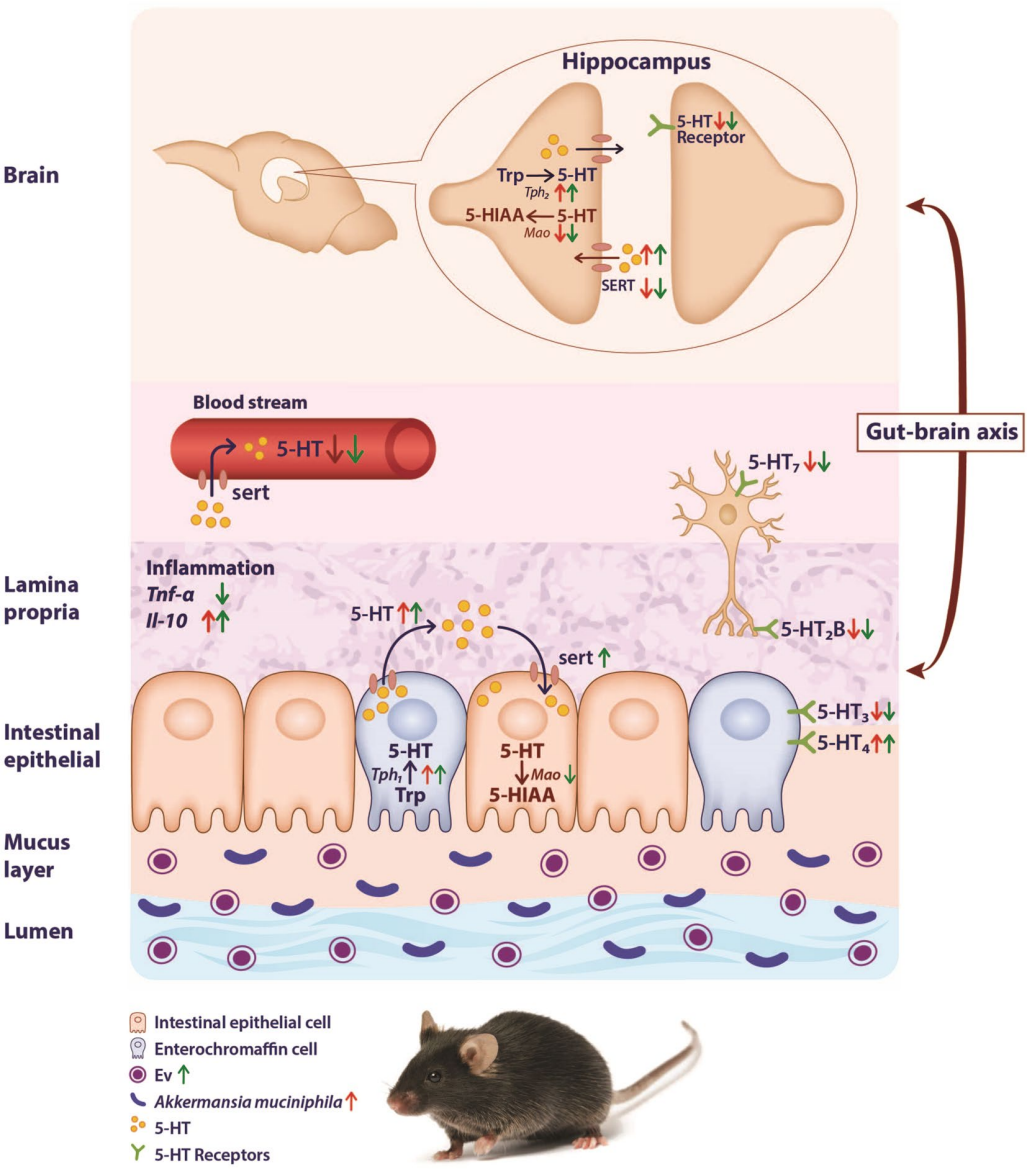
The oral administration of *A. muciniphila* and its EVs affected the serotonin pathway through the gut-brain axis in mice. This figure shows major intermediates, enzymes, and transporters in serotonin synthesis and clearance. Colonic and hippocampal serotonin synthesis increased through interactions with the bacterium and its EVs and upregulation of *Tph1* gene in the colon and *Tph2* gene in the hippocampus. Also, the increased serotonin availability in the colon of mice increased SERT expression in both groups. Conversely, the SERT expression decreased in the hippocampus of both groups. The *Mao* gene expression reduced following EV treatment in the colon and following both treatments in the hippocampus. Treatments with *A. muciniphila* and its EVs affected colonic inflammation and inflammation-related genes. Compounds: 5-HT = serotonin; Enzymes & transporters: *Tph* = Tryptophan hydroxylase, SERT = Serotonin transporter, *Mao* = Monoamine oxidase.

The mRNA expression of the *Tph1* gene was significantly higher in the EV (P = 0.0001) groups compared to the control ones (Fig. 6B). In the cell line, the EVs induced a higher level of *Tph1* expression, compared to the *A. muciniphila* group. Similar to the in vivo results, *A. muciniphila* and EVs induced the upregulation of the *Slc6a4* gene in the cell line (P = 0.0001 and P = 0.0001, respectively). Meanwhile, the bacterium exerted a more significant effect than EVs (Fig. 6C). However, the mRNA expression of the *Mao* gene was unaffected by the bacterium and EV treatments (Fig. 6D). Overall, both the bacterium and EVs had positive effects on the serotonin level in vitro (Fig. 7).

## Discussion

Recent research has clearly shown that serotonin, as a neurotransmitter in the gut-brain axis, can be modulated by intestinal microbiota or their metabolites in the GI tract, in this way, gut microbiota plays a crucial role in peripheral and central serotonin pathways^7,8,32^. There is limited information about the emerging link between the gut microbiota and the serotonergic system across the microbiota-gut-brain axis^33^. The present study showed that *A. muciniphila* and its EVs could affect the serotonergic system in the colon and hippocampus of mice.

Similar to some previous studies, which revealed that the gut bacteria could induce serotonin biosynthesis from EC cells in the colon^8^, our results also highlighted that *A. muciniphila* could stimulate serotonin biosynthesis in the colon. In this regard, Nzakizwanayo et al. showed that probiotic *Escherichia coli* strain Nissle 1917 enhanced the serotonin bioavailability in the gut tissues^34^. However, in our study, after 5 weeks of *A. muciniphila* administration, the production of serotonin increased in the hippocampus of mice. Our finding shows that bacteria can be involved in the brain serotonin synthesis. There was also a positive correlation between the gut microbiota-derived metabolites and brain serotonin levels; nevertheless, we did not have any evidence supporting this speculation in our study. Moreover, Tian et al. revealed that the gut *Bifidobacterium breve* CCFM1025 could affect the brain function and exert beneficial psychotropic effects^35^. Therefore, probiotic modulation of the gut microbiota composition may propose a novel treatment for brain dysfunctions^35^; our observations are in line with the mentioned result.

About 90% of the serotonin in the body is synthesized in the gut, while the rest is synthesized in the brain. The brain and gut serotonin systems are separated by the blood–brain barrier (BBB), and it should be noted that serotonin cannot cross this barrier^36,37^. Peripheral serotonin is an endocrine factor, which regulates the metabolic function of several tissues, except the brain tissue. Notably, *A. muciniphila*-derived EVs similar to the bacterium itself could increase the serotonin level in the colon and hippocampus of mice. Similarly, EVs may have exerted these effects through different mechanisms, which may be related to its ability to cross the BBB or might be linked to signal transduction pathways, however, we have not experimentally evaluated whether these EVs cross the BBB. Therefore, preliminary experiments are required to be carried out on EVs. These findings may indicate the potential role of EVs as postbiotic agents, acting as vehicles for bidirectional communication between the gut and the brain.

Dysregulation of the serotonin system is also associated with intestinal inflammation, which acts as a link between metabolic homeostasis and serotonin pathways^38,39^. Therefore, further research is needed to understand the relationship between the serotonergic system and the maintenance of homeostasis in the gut.

It is not clear whether synthesized serotonin in the colon is associated with inflammation. Strong evidence suggests that modulation of selective serotonergic receptors or serotonin signal termination (via serotonin reuptake transporters) can modify the risk and severity of intestinal inflammation^40^. Therefore, in this study, to evaluate inflammation, we examined the gene expression of the proinflammatory cytokine, *Tnf*-α, and anti-inflammatory cytokine, *Il-10,* besides the histology of colon tissue, and by examining these inflammatory parameters, it seems that *A. muciniphila* and its EVs may have not caused inflammation in the colon, despite affecting serotonin production in the colon. In same vein, Ashrafian et al., indicating the anti-inflammatory activities of *A. muciniphila* and its EVs^25^, our findings also confirmed the beneficial effects of this bacterium as a next-generation probiotic and its EVs as postbiotic agents.

We analyzed the effects of *A. muciniphila* and its EVs on major genes involved in the serotonergic system, contributing to serotonin synthesis, reuptake, metabolism, and receptor binding. The colonic mRNA level of the *Tph1* gene (a rate-limiting enzyme for serotonin biosynthesis in EC cells) increased following both treatments. Overall, our findings are in line with the results reported by Yano et al., which showed that specific microbial metabolites could increase serotonin synthesis by upregulating *Tph1* gene expression in EC cells^8^. In comparison with the bacterium, *A. muciniphila*-derived EVs exerted more significant effects on *Tph1* gene expression in the submucous-mucous layer of the colon. Since *Tph1* is an intracellular enzyme, we hypothesized that EVs could have greater effects on the expression of this gene by accessing the cells.

Similarly, Reigstad et al*.* reported that microbial products, such as SCFAs, can augment *Tph1* and 5-HT synthesis in EC cells^7^. Similar to in vivo studies, EV treatment induced the overexpression of the *Tph1* gene in the cell line. Despite an increase in *Tph1* gene expression, no significant changes were observed in the serotonin level following EV exposure; this difference can be explained by post-transcriptional modifications. Next, we investigated the potential changes in the expression of *the Tph2* gene, which is another TPH isoform, involved in 5-HT biosynthesis in the brain. In the hippocampus, we observed an increase in *Tph2* gene expression in both groups. These results support the hypothesis that both treatments could improve 5-HT synthesis by upregulating *Tph2* gene expression in the brain.

Moreover, we found that *A. muciniphila*-derived EVs induced more biological impact on *Tph2* gene expression, which might be related to BBB, as EVs, may pass through this barrier and affect the expression of *Tph2* gene. The results of this study are in line with those reported by Li et al., which showed that *Bifidobacterium longum* and *L. rhamnosus* significantly increased the levels of serotonin and *Tph2* genes in the brain^41^. In another study by Borrelli et al., *L. rhamnosus* IMC 501 strain significantly upregulated the expression of *the Tph2* gene in the brain of zebrafish^42^.

Until now, there have been no studies investigating the effect of EVs on the serotonin system in the brain. Our results clarified the effects of *A. muciniphila* and its EVs on the reuptake and clearance of serotonin in both the gut and the brain. The serotonin transporter (i.e., SERT), which is encoded by the *Slc6a4* gene, plays an incomparable role in the inactivation of serotonin through the uptake of released 5-HT from the interstitial space in the lamina propria into the neighboring enterocytes of the gut and presynaptic neurons of the brain. However, any dysfunction in SERT causes an imbalance in the level of 5-HT and affects the intestinal function, including the intestinal fluid secretion, intestinal motility, and GI perception, decrease expression of SERT in the gut is implicated in subsets of irritable bowel syndrome (IBS), therefore SERT plays a pivotal role in IBS pathogenesis^43,44^. In the present study, we examined the expression of *Slc6a4* mRNA in the colon. Our results indicated that in EV group, SERT was seemingly upregulated. In this regard, Cao et al. reported that administration of *L. rhamnosus* GG supernatant (LGG-s) increased the SERT mRNA expression in the intestinal tissue of rats with post-infectious IBS^45^. In terms of enhance SERT function, Martín et al. showed that *F. prausnitzii* and its supernatant restored the level of SERT to normal levels in a murine model with chronic low-grade inflammation, which highlights the useful effect of *F. prausnitzii* and suggests a higher exploration capability and attention as a probiotic therapeutic agent for improvement of the gut inflammatory disorders^46^.

Also, a recent study revealed that the increase of SERT function, as a major regulator of 5-HT signaling, may have greater effects on the homeostasis of the serotonergic system. Accordingly, practical suggestions were proposed for the treatment of various pathophysiological disorders in humans.

Since the plasma serotonin is derived from the gut, any impairment of the SERT function can alter the plasma serotonin level. Most previous studies have shown that reduction of SERT function is associated with an increase in the plasma level of 5-HT and is involved in IBS, celiac disease, and diarrheal disease^47,48^. Similarly, our observations showed that the administration of *A. muciniphila* and its EVs led to the reduction of serum serotonin. In line with the abovementioned results, we showed that SERT expression increased in both groups. Also, both the bacterium and its EVs stimulated the expression of the *Slc6a4* gene not only in mice, but also in the Caco-2 cell line.

Despite the differences between in vivo and in vitro conditions, both groups showed similar results under these conditions; it can be concluded that they had direct effects on the expression of the *Slc6a4* gene. Moreover, the expression of the *Slc6a4* gene reduced in the brain and consequently, we observed the independent effects of the bacterium and its EVs in the gut and the brain. Administration of the bacterium and its EVs increased the synthesis of serotonin and decreased the reuptake and expression of the *Mao* gene in the brain; accordingly, they may be considered as serotonin modulator in serotonergic pathways. The ENS and CNS systems play crucial roles in various physiological functions of the body^49^. Serotonin, by interacting with a variety of serotonergic receptors, plays a crucial role as an active mediator across this axis^50^. Given the complex structure of the serotonergic system, one of the strategies, which can help us understand its function, is to evaluate receptors, such as *Htr1*, *Htr*2, *Htr*3, *Htr*4, *Htr*5, *Htr*6, and *Htr*7. Therefore, in addition to understanding the effects of the bacterium and its EVs on the major elements of intestinal 5-HT signaling (e.g., 5-HT synthesis, release, receptor expression, and reuptake capacity), we evaluated the gene expression of different serotonin receptors in the colon and hippocampus of mice. In the colon, the expression of *Htr2B*, *Htr3B,* and *Htr7* genes decreased, while the expression of *the Htr4* gene increased following both treatments. Changes in the expression of these receptors can be explained by feedback regulation through reciprocal binding between serotonin and different receptors.

Since *5- Htr3* and *5*- *Htr4* genes are important secreted mediators in the intestine, they have been more examined in the literature^12,14,51^. These investigations showed that the agonists and antagonists of these receptors have pharmaceutical applications^52,53^. Yogesh et al. demonstrated that after incubation of colonoids with acetate, as a gut microbiota metabolite, the expression of 5-HT3 receptor significantly decreased in germ-free (GF) mice^54^, which is consistent with our results. Also, a previous study reported that agonists of 5-HT4 receptors, which are found on the apical side of epithelial cells in the gut, activate the release of 5-HT from EC cells^55^.

Our findings also confirmed that the upregulation of *Htr4* gene expression might activate 5-HT content in the colonic mucosa of mice following treatment with both *A. muciniphila* and its EVs. Yano et al. showed that colonization of GF mice with spore-forming bacteria elevated the colonic serotonin level and improved the GI motility by activati*ng Htr4* enteric neurons^8^. Moreover, recent research has shown that the expression of serotonin receptor genes, involved in the serotonergic system, is variable and unclear. Therefore, increasing attention has been paid to recognize the function of these receptors and to determine the impact of the gut microbiota on the serotonergic system.

The effects of the gut microbiota and their metabolites on the serotonergic system of the brain still need to be studied. In the current study, we reported the effects of *A. muciniphila* and its EVs on the expression of serotonin receptors in the hippocampus of mice for the first time. Besides evaluating the impact of change in serotonin, targeting specific 5-HT receptors should also be considered as a treatment strategy. However, we found that the expression of *Htr1A, Htr2A, Htr5,* and *Htr6* genes decreased by both treatments, indicating a significant association between the administration of the bacterium and its EVs and the serotonergic system in the brain.

One of the most important receptors, involved in serotonin signaling in the brain, is 5HT1A; therefore, increases in 5-HT1A receptor binding were significantly correlated to increasing anxiety^56^. In this regard, Szklany et al. showed that supplementation of prebiotics, e.g., short-chain galacto-oligosaccharides (scGOS) and long-chain fructo-oligosaccharide (lcFOS), decreased the mRNA expression of *Htr1A* gene in the prefrontal cortex and also reduced anxiety-like and repetitive behaviors in mice^57^. This finding supported the hypothesis that specific probiotics or prebiotics, by affecting serotonin signaling, can have potential behavioral benefits.

Overall, an imbalance in either the gut or brain serotonergic system may contribute to the pathogenesis of various disorders. Therefore, maintenance of intestinal homeostasis via probiotic-based modulation can balance the serotonergic system and improve a wide range of disorders, with possible beneficial effects on the host health. So far, most previous studies have examined the association between the intestinal microbiota and the serotonergic system in the gut, while our results highlighted the impact of the microbiota-gut-brain axis on regulating the serotonergic system. However, further investigations are needed to understand the precise mechanism of intestinal bacteria's effects on the serotonergic system across the gut-brain axis.

In the present study, EVs, had strong effects on the serotonergic system, compared to the bacterium in both the gut and the brain. Therefore, they may be considered as key mediators between the ENS and CNS and are crucial for the development of microbiome-targeted therapeutic approaches to improve psychiatric diseases. The main advantage of using EVs over bacteria is that they are non-viable and can be controlled in the body by modifying their dosage. Since EVs are nanosize and non-replicative particle, they show inherent stability under different body conditions and are more biocompatible than bacteria, however, our experiments did not provide evidence to support that these EVs survived the transit through the gastrointestinal tract following oral administration. Moreover, the use of probiotic-derived bioactive molecules as postbiotics (e.g., EVs) can have potential therapeutic benefits, such as the maintenance of intestinal homeostasis and reduction of the risk of live bacteria administration. Carefully controlled studies on the host health are needed to evaluate the greater impact of 5-HT system modulation in response to treatment with agents, such as EVs.

In conclusion, since microbiota is critical for the host’s serotonergic system, our results showed that the bacterium and its EVs could modulate serotonin signaling/metabolism through the gut-brain axis in mice. It can be suggested that supplementation with a probiotic is sufficient to induce changes in bidirectional signaling between the microbiota and the brain. Although we used a powerful approach in this study, there are some limitations, and further experiments are recommended to understand the interactions of the microbiota-gut-brain axis. Overall, our results suggested the practical use of probiotic treatment for the development of microbiota-based therapeutic strategies for psychiatric diseases.

## Supplementary Information

Supplementary Information.
