# Supplementary material for: Modulation of serotonin signaling/metabolism by Akkermansia muciniphila and its extracellular vesicles through the gut-brain axis in mice
Source: Sci Rep. 2020 Dec 17;10:22119. doi: 10.1038/s41598-020-79171-8 (PMC7747642; doi:10.1038/s41598-020-79171-8)
Supplement: Supplementary file 1 — Supplementary Information. [file 41598_2020_79171_MOESM1_ESM.docx]

**Modulation of serotonin signaling/metabolism by *Akkermansia muciniphila* and its extracellular vesicles through the gut-brain axis in mice**

Rezvan Yaghoubfar^1,2^, Ava Behrouzi^1,2^, Fatemeh Ashrafian^1,2^**,** Arefeh Shahryari^1,2^, Hamid Reza Moradi^3^, Samira Choopani^4^, Shima Hadifar^1,2^, Farzam Vaziri^1,2^, Seyed Ali Nojoumi^1,2^, Abolfazl Fateh^1,2*^, Shohreh Khatami^5*^, Seyed Davar Siadat^1,2^

**Table 1.** The oligonucleotide sequence of primers for the cell line target genes in mice. The set of primers used for *in vivo* experiments is represented by the letter *m* in the table.

| Primer name | Forward primer | Reverse primer | Product size (bp) | Reference |
| --- | --- | --- | --- | --- |
| m*-β-actin* | GAGGTATCCTGACCCTGAAGTA | CACACGCAGCTCATTGTAGA | 104 | (1) |
| m*-Rpl-13A* | AGCCTACCAGAAAGTTTGCTTAC | GCTTCTTCTTCCGATAGTGCATC | 129 | (2) |
| m*-Tph1* | ACTGCGACATCAGCCGAGAA | CGCAGAAGTCCAGGTCAGAAATC | 162 | (3) |
| m*-Tph2* | GAGCAGGGTTACTTTCGTCCATC | AAGCAGGTCGTCTTTGGGTCA | 91 | (4) |
| m*-Htr1aa* | CCGTGAGAGGAAGACAGTGAAGA | GGTTGAGCAGGGAGTTGGAGTAG | 176 | (4) |
| m*-slc6a4a* | TATCCAATGGGTACTCCGCAG | CCGTTCCCCTTGGTGAATCT | 110 | (5) |
| m*-Mao* | GGAGAAGCCCAGTATCACAGG | GAACCAAGACATTAATTTTGTATTCTGAC | 113 | (6) |
| m*-Htr2B* | TTATGCGAAGAATGTCCT | TTGAGAGTGGTCTGATTG | 175 | This study |
| m*-Htr2A* | CGAAGCCTCGAACTGGACAAT | CCGCAATGGTGAGAATAATCACG | 125 | This study |
| m*-Htr3B* | CTGTCTACCTGGACCTTTGCG | AACTCATCGTTCCAAACCTCTC | 103 | This study |
| m*-Htr4* | GATGCTAATGTGAGTTCCAACGA | CAGCAGGTTGCCCAAGATG | 105 | This study |
| m*-Htr5* | CTCTACCTCTGCCTCTGTT | GTCCTTCGTTCTGTTCACTT | 192 | This study |
| m*-Htr6* | CCAACTCAGATTCAGACTCAG | GTATCTCAGGCTCCACAGA | 159 | This study |
| m*-Htr7* | TGCGGGGAGCAGATCAACTA | GACAAAGCACACCGAGATCAC | 120 | (7) |
| m*-Il-10* | GCACTACCAAAGCCACAAG | AGTAAGAGCAGGCAGCATAG | 85 | This study |
| m*-Tnf-α* | AACAACTACTCAGAAACACAAG | GCAGAACTCAGGAATGGA | 130 | This study |
| *GAPDH* | GGAGCGAGATCCCTCCAAAAT | GGCTGTTGTCATACTTCTCATGG | 197 | (8) |
| *Tph1* | TGCAAAGGAGAAGATGAGAGAATTTAC | CTGGTTATGCTCTTGGTGTCTTTC | 114 | (9) |
| *Slc6a4* | TTCAACAACAACTGCTACCA | ACACATCTTCATTCCTCATCTC | 127 | This study |
| *Mao* | TTCAGGACTATCTGCTGCCAA | GGTCCCACATAAGCTCCACC | 147 | (10) |

**References**

1. Zhang, Z. et al. CEACAM-1 promotes myocardial injury following coxsackievirus infection by regulating the coxsackievirus-adenovirus receptor. *Medicine*. **98**, e15629 (2019)

2. Zhang, Y., Rogers, HM., Zhang, X. & Noguchi, CT. Sex difference in mouse metabolic response to erythropoietin. *FASEB. J*. **31**, 2661-2673 (2017).

3. Hata, T. et al. Regulation of gut luminal serotonin by commensal microbiota in mice. *PLoS One*. **12**, e0180745 (2017).

4. Ishikawa, C. et al. Effects of gravity changes on gene expression of BDNF and serotonin receptors in the mouse brain. *PloS one*. **12**, e0177833 (2017).

5. Sada, A. et al. Defining the cellular lineage hierarchy in the interfollicular epidermis of adult skin. *Nat Cell Biol.* **18**, 619-31 (2016).

6. Lee, AK. et al. Effect of high-fat feeding on expression of genes controlling availability of dopamine in mouse hypothalamus. *Nutrition*. **26**, 411-422 (2010).

7. Zhong, H. et al. Effects of medial prefrontal cortex 5-HT7 receptor knockdown on cognitive control after acute heroin administration. *Brain. Res*. **1678**, 419-431 (2018).

8. Shao, Y. et al. Zinc enhances intestinal epithelial barrier function through the PI3K/AKT/mTOR signaling pathway in Caco-2 cells. *J. Nutr. Biochem*. **43**, 18-26 (2017).

9. Van Lelyveld, N., Ter Linde, J., Schipper, M. & Samsom M. Regional differences in expression of TPH-1, SERT, 5-HT(3) and 5-HT(4) receptors in the human stomach and duodenum. *Neurogastroenterol. Motil*. **19**, 342-348 (2007).

10. Ooi, J., Hayden, MR. & Pouladi, MA. Inhibition of excessive monoamine oxidase A/B activity protects against stress-induced neuronal death in Huntington disease. *Mol. Neurobiol*. **52**, 1850-1861 (2015).
